# Seasonal and Differential Sesquiterpene Accumulation in *Artemisia annua* Suggest Selection Based on Both Artemisinin and Dihydroartemisinic Acid may Increase Artemisinin *in planta*

**DOI:** 10.3389/fpls.2018.01096

**Published:** 2018-08-13

**Authors:** Jorge F. S. Ferreira, Vagner A. Benedito, Devinder Sandhu, José A. Marchese, Shuoqian Liu

**Affiliations:** ^1^US Salinity Laboratory, Riverside, CA, United States; ^2^Division of Plant and Soil Sciences, West Virginia University, Morgantown, WV, United States; ^3^Biochemistry and Plant Molecular Physiology Laboratory, Agronomy Department, Federal University of Technology–Paraná, Pato Branco, Brazil; ^4^Department of Tea Science, College of Horticulture and Hardening, Hunan Agricultural University, Changsha, China

**Keywords:** seasonal sesquiterpene accumulation, sesquiterpene-based selection, sesquiterpene seasonal peak, high-DHAA germplasm selection, different chemotypes

## Abstract

Commercial *Artemisia annua* crops are the sole source of artemisinin (ART) worldwide. Data on seasonal accumulation and peak of sesquiterpenes, especially ART in commercial *A. annua*, is lacking while current breeding programs focus only on ART and plant biomass, but ignores dihydroartemisinic acid (DHAA) and artemisinic acid (AA). Despite past breeding successes, plants richer in ART are needed to decrease prices of artemisinin-combination therapy (ACT). Our results show that sesquiterpene concentrations vary greatly along the growing season and that sesquiterpene profiles differ widely among chemotypes. Field studies with elite Brazilian, Chinese, and Swiss germplasms established that ART peaked in vegetative plants from late August to early September, suggesting that ART is related to the photoperiod, not flowering. DHAA peaks with ART in Chinese and Swiss plants, but decreases, as ART increases, in Brazilian plants, while AA remained stable through the season in these genotypes. Chinese plants peaked at 0.9% ART, 1.6% DHAA; Brazilian plants at 0.9% ART, with less than 0.4% DHAA; Swiss plants at 0.8% ART and 1% DHAA. At single-date harvests, seeded Swiss plants produced 0.55–1.2% ART, with plants being higher in DHAA than ART; Brazilian plants produced 0.33–1.5% ART, with most having higher ART than DHAA. Elite germplasms produced from 0.02–0.43% AA, except Sandeman-UK (0.4–1.1% AA). Our data suggest that different chemotypes, high in ART and DHAA, have complementary pathways, while competing with AA. Crossing plants high in ART and DHAA may generate hybrids with higher ART than currently available in commercial germplasms. Selecting for high ART and DHAA (and low AA) can be a valuable approach for future selection and breeding to produce plants more efficient in transforming DHAA into ART *in planta* and during post-harvest. This novel approach could change the breeding focus of *A. annua* and other pharmaceutical species that produce more than one desired metabolite in the same pathway. Obtaining natural variants with high ART content will empower countries and farmers who select, improve, and cultivate *A. annua* as a commercial pharmaceutical crop. This selection approach could enable ART to be produced locally where it is most needed to fight malaria and other parasitic neglected diseases.

## Introduction

Active pharmaceutical ingredients (APIs) based on artemisinin (ART), such as artemether and artesunate, are key components of the most effective antimalarial drugs. The Nobel Assembly at Karolinska Institute awarded the Nobel Prize in Physiology or Medicine to Prof. Youyou Tu in 2015 in recognition of her early work in the late 1960s and 70s that culminated with the discovery of ART, the most effective natural anti-malarial medicine after quinine. Currently, all ART that is used as raw material for the production of artemisinin-combination therapy (ACT) is obtained from the plant *Artemisia annua* (Family: Asteraceae). Although other plants and microorganisms have been engineered with ART-pathway genes, tobacco was only able to produce artemisinic acid (AA) at 0.12% of leaf dry weight (DW) (Fuentes et al., 2016), and ART at less than 0.0007% of leaf DW (Farhi et al., 2011); transgenic moss produced ART at 0.021% of leaf DW (Khairul Ikram et al., 2017). These yields are 47 (moss) to 1,400 times (tobacco) less than an *A. annua* plant that produces 1% ART. Also, moss is not a feasible alternative considering its low biomass yield. Although baker's yeast produced 25 g of AA per liter of culture (Paddon et al., 2013), derivatization of AA to ART is further needed, making the process economically unfeasible compared to plant-based ART (Peplow, 2016). The low yields of ART in tobacco may be in part due to the fact that ART is phytotoxic (Duke et al., 1987) and must be stored extracellularly, inside epicuticular spaces of glandular trichomes (Duke et al., 1994; Ferreira and Janick, 1995b). Although other Artemisia species can produce ART (Mannan et al., 2010), their ART shoot concentrations are not large enough to justify commercialization. The no-cost/no-profit price of semi-synthetic ART by Sanofi is estimated to range from US$350 to 400 kg^−1^, which is well above the US$250 kg^−1^ for the naturally-produced ART (Peplow, 2016). Although heterologous systems are valuable to improve the knowledge of the ART pathway, more efficient ways to stabilize market prices and reduce ACT costs are urgently needed. Rather than pursuing ART production in heterologous systems, more viable approaches to increase plant-based ART supply should focus on: (1) breeding plants that are higher in ART than the currently reported average of approximately 1.5% ART, (2) producing cultivars that are less variable in plant-to-plant ART concentrations, (3) producing cultivars that are higher in DHAA and able to convert DHAA into ART more efficiently, (4) improving ART commercial extraction to over 70% efficiency, and (5) recycling DHAA from ART commercial waste to produce more ART.

Since the initial plant screenings by the Chinese government in the late 1960s, plants have been selected only for their high biomass and ART leaf content. In 1985, Chinese accessions were reported to range from 0.01 to 0.5% ART, with plants from the Sichuan and Chongqing provinces being the highest in ART (Klayman, 1985), and those north of the Huaihe river bank reported to have 0.1% ART or less (Li et al., 2017). Only a few breeding programs managed to increase ART shoot concentration from 0.5 to 1.5% or higher. Because *A. annua* has a high degree of self-incompatibility (allogamy) that precludes self-pollination, and the word “hybrid” often used does not denote true hybrids generated from homozygous parents, but rather the F_1_ progeny of two distinct, highly heterozygous, parents. The oldest breeding program with published reports in English and French is from the company Mediplant (Conthey, Switzerland). In the early 1990s this program produced several crossings that generated plants with over 1% ART (Debrunner et al., 1996; Magalhães et al., 1999). Later, Mediplant reported a new line named “Hybrid 1” with up to 1.8% ART and 2.9 tons of dry leaves ha^−1^ (Simonnet et al., 2008), but we are not sure whether “Hybrid 1” was ever available commercially or to breeding programs destined to generate high-ART plants for humanitarian purposes. The Brazilian breeding program, in collaboration with Mediplant, developed hybrids named “CPQBA,” the acronym (in Portuguese) for the Multidisciplinary Center for Chemical and Biological Research (Campinas, Brazil) with seeds that sold for US$40 g^−1^, with 12–15 thousand seeds g^−1^. Despite the fact that plants from both Mediplant and CPQBA selections were late flowering in the environment where they were selected (Nicolas Delabays, personal communication), they flowered prematurely and produced little biomass when planted close to the equator (5–7° latitude) due to a short day length that inhibit vegetative development and accelerated the reproductive stage (Ferreira et al., 2005). Selected plants from this Swiss (Mediplant) cultivar named Artemis® produced from 0.65 to 1.9% ART when cultivated in an Appalachian Gilpin soil (Beaver, WV, 37°44′N 81°8′W) from May to August in a Quonset greenhouse under a mild potassium stress (Ferreira, 2007). Most recently, the cross “Hyb8001r” was developed and introduced by the Centre for Novel Agricultural Products (CNAP) in the UK, which is now commercialized by East-West Seed International. In field trials worldwide, “Hyb8001r” produced shoot ART up to 1.44% (g/100 g DW) and up to 4.4 tons/ha of dry leaf biomass, with a theoretical ART yield of 54 kg/ha (Suberu et al., 2016). In their work, “Hyb8001r” is referred to as CNAP8001, with seeds available to growers linked to the ACT raw material supply chain (https://www.artemisiaf1seed.org/hyb8001r/).

**Figure 1:**
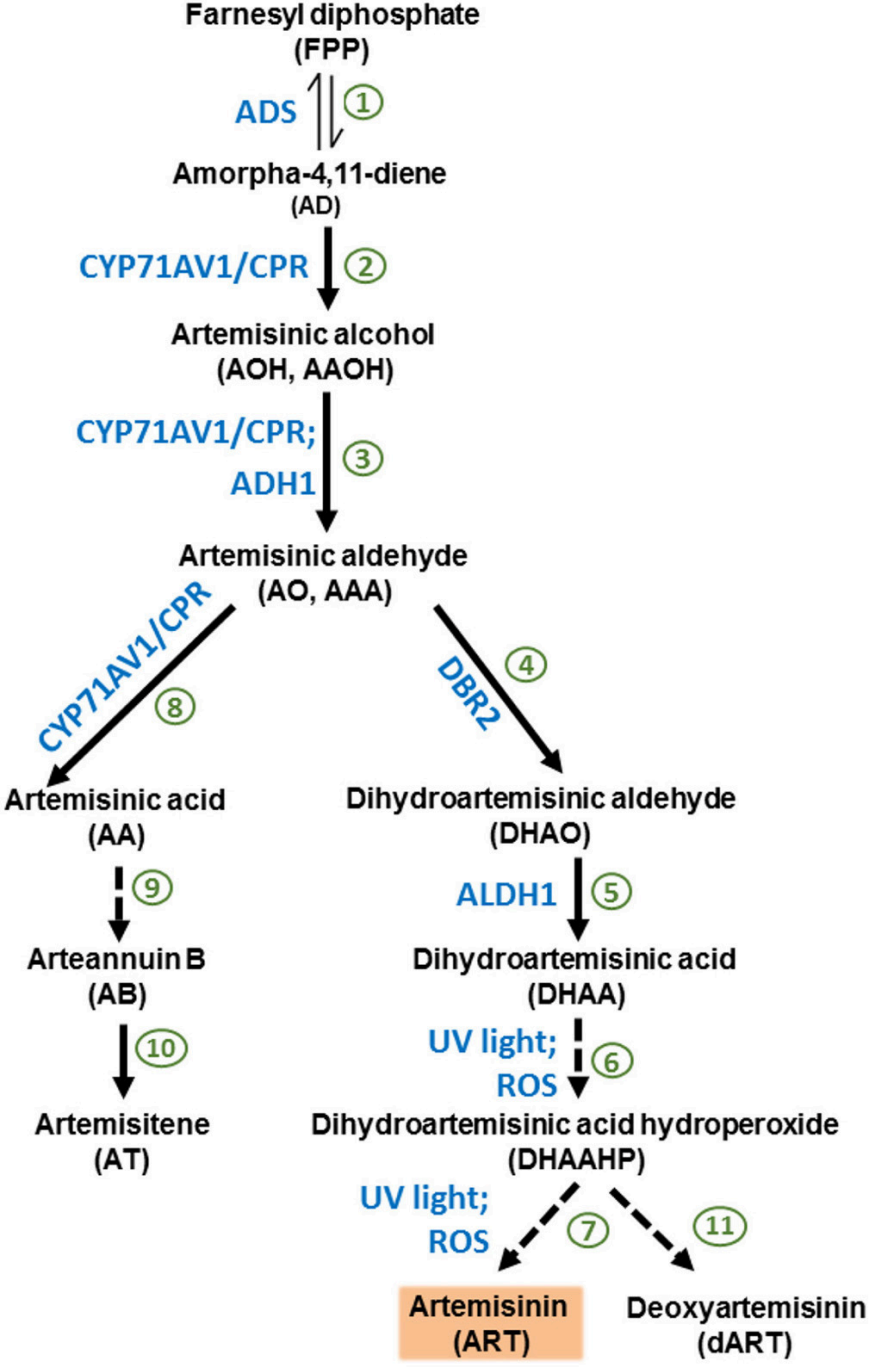
Biosynthetic pathway of artemisinin starting from farnesyl diphosphate (FPP). 1. Amorpha-4,11-diene synthase (ADS) synthesizes the first committed product of the artemisinin pathway, AD. 2. Cytochrome P450 monooxygenase CYP71AV1 oxidizes AD to AA in three successive reactions (2, 3, and 8). CYP71AV1 is the main rate-limiting enzyme of the committed artemisinin pathway. Cytochrome P450 reductase (CPR) restores the active state of CYP71AV1. 3. AO can be produced by CYP71AV1/CRP enzymatic system, and alcohol dehydrogenase 1 (ADH1) can also make AO using AOH as a substrate. 4. AO is utilized by the enzyme artemisinic aldehyde Δ11(13) reductase (DBR2), which generates DHAO, as well as the third reaction of CYP71AV1 (7), which generates AA. 5. Aldehyde dehydrogenase 1 (ALDH1) converts DHAO to DHAA. 6–7. The conversion of DHAA into ART through the intermediate DHAAHP is postulated to be non-enzymatic, but induced only by ultraviolet light and oxygen. 8. The third reaction of CYP71AV1 converts AO into AA, diverting the pathway from producing ART and DHAA. 9. AA can be converted non-enzymatically into AB. 10. AB can be converted into AT. 11. DHAAHP may also generate DART. Once extracted from plants and purified, ART is derivatized into dihydroartemisinin (dART), then into other antimalarial drugs (e.g., artemether and artesunate), which the body metabolizes to the bioactive DART.

The literature provides evidence that *A. annua* plants accumulate not only ART, but also its biosynthetic precursor DHAA and AA (Wallaart et al., 2000; Ferreira, 2007; Ferreira and Luthria, 2010). After artemisinic aldehyde (AO), the ART pathway diverts into either AA, arteannuin B, and artemisitene (AT) or into DHAA, dihydroartemisinic hydroperoxide (DHAHP), and ART (Kjær et al., 2013; Bryant et al., 2015) (Figure 1). Despite the fact that AA can be the main sesquiterpene produced (over 1% of leaf DW) by certain chemotypes of *A. annua* (Ro et al., 2006), the chemotype used for commercial purposes produce mainly ART, with DHAA as its main precursor (Wallaart et al., 1999b; Brown and Sy, 2004; Ferreira and Luthria, 2010). Contrary to previous reports of that *in vitro* photooxidative conversion of DHAA into ART could occur with almost 27% efficiency in organic solvents and with the presence of chlorophyll *a* (Acton and Roth, 1992; Wallaart et al., 1999a). Recent work suggests enzymatic action in the final stages of the pathway (Zhu et al., 2014; Bryant et al., 2015).

It was originally postulated that ART was produced and sequestered in glandular trichomes of leaves (Duke et al., 1994) and flowers (Ferreira and Janick, 1995a,b); and that the isolation of enzymes from these trichomes would confirm this hypothesis (Ferreira and Janick, 1995a). Since then, ART biosynthetic enzymes have been isolated from glandular trichomes (Olofsson et al., 2012; Tan et al., 2015; Chen et al., 2017). However, although the pathway produces AA, DHAA, and ART simultaneously, the two main chemotypes found in the literature either accumulate mainly AA (Elhag et al., 1992; Ro et al., 2006) or mainly DHAA and ART (Wallaart et al., 1999b, 2000; Ferreira, 2008). Detailed experiments based on the use of synthetically-produced radio-labeled precursors fed to plants also concluded that, compared to AA, DHAA is more cost-effective to produce ART in genetically-engineered yeast (Brown, 2010). A two-step auto-photooxidation step is postulated to occur in plants to convert DHAA into ART while plants senesce, or are dried under oven, shade, or sun (Brown and Sy, 2004; Ferreira and Luthria, 2010). Sun drying for 1–3 weeks was the most efficient way to convert DHAA into ART (over 90%), whereas forced-air oven drying (45°C from 12 to 16 h) only achieved a 40% conversion (Ferreira and Luthria, 2010). These findings suggest that drying plants in a forced-air oven illuminated with UV light may convert DHAA into ART more efficiently than in a dark oven. The average content of ART in plants used for industrial extraction has been reported to be 0.7% based on leaf dry weight (Malcolm Cutler, personal communication). However, ART yields can be maximized if the plants are harvested at the time ART reaches its seasonal peak, which is prior to flowering (Delabays et al., 2001; Ferreira, 2008). Swiss plants field-cultivated in West Virginia and harvested toward the end of their vegetative stage (no flowers), in early September, produced 0.7% ART and an average of 450 g dry leaves plant^−1^, or 4.5 tons ha^−1^ for a plant density of 1 plant m^−2^ (Ferreira, 2007).

*A. annua* plants increased ART production in response to abiotic stresses, such as potassium deficiency (Ferreira, 2007), drought (Marchese et al., 2010), post-harvest drying (Ferreira and Luthria, 2010), senescence (Lommen et al., 2007), and salinity (Qureshi et al., 2005; Qian et al., 2007; Yadav et al., 2017). Interestingly, environmental stresses such as drought, wound, and cadmium (Xiao et al., 2016), and application of the hormones JA and cytokinin (Maes et al., 2011) increased trichome density in *A. annua*. Trichome development and ART biosynthesis have been linked through the transcription factor TAR1 and its role in upregulating ADS, CYP71AV1, and ART biosynthesis (Tan et al., 2015) and several other genes, reviewed elsewhere (Xiao et al., 2016). The literature also suggests that reactive oxygen species (ROS) triggered by stress may lead to the transformation of DHAA into ART, although that has not yet been shown *in planta* through direct correlation between quantified ROS build-up and the increased conversion of DHAA into ART. Also, although potassium deficiency stress increased ART leaf concentration by 75%, it had no apparent effect on the concentrations of either DHAA and AA (Ferreira, 2007). Thus, the simple effect of photooxidation seems more plausible at this point, although it is unknown how DHAA is converted into ART as the plant metabolism shuts down during senescence and drying. For instance, compared to freeze dried sub-samples, shade, oven, and sun dried shoots had significantly higher concentrations of ART and decreased concentrations of DHAA and AA, and sun drying was more efficient in converting DHAA into ART (Ferreira and Luthria, 2010). Although it is currently debatable whether the last steps of the pathway (leading from DHAA to ART and deoxyartemisinin (dART) through dihydroartemisinic acid hydroperoxide (DHAAHP) are enzymatic or not, if selection and breeding focus exclusively on optimizing ART yields (and neglects DHAA), the potential to increase ART production *in planta* through the conversion of DHAA into ART during post-harvest drying will be wasted.

In order to validate their production potential, promising elite germplasm should be tested in areas with similar edaphoclimatic conditions of potential commercial production. A new cultivar (“Artemis®”) developed in Conthey, Switzerland (46°13′N 7°17′E, elevation 485 m above sea level - asl), and another (“A3”) developed by the University of Campinas, Brazil (22°48′S, 47°07′W, 749 m asl) have been tested in Kenya, Tanzania, and Nigeria, and produced from 0.7 to over 1% ART DW. However, a Chinese genotype from Chongqing, China (29.43°N, 106.91°E, 238 m asl) was grown only in China and Vietnam until its field trials in West Virginia (37°45′N 80°50′W, 890 m asl) reported here. Further selections of the Brazilian cultivar better adapted to lowland humid tropics resulted in plants with higher ART (1% w/w) and leaf dry biomass (3 ton/ha) than the Chinese, Indian, and U.S. clones (0.4–0.5% ART, 1.5–2 ton/ha of dry leaf biomass) in Calabar, Nigeria (4.96°N 8.3°E, 50 m asl) (Brisibe et al., 2012). To our knowledge, there are no current breeding programs engaged in producing *A. annua* genotypes that are rich in both ART and DHAA, or with a less variable ART yield from plant to plant.

The aims of this work are to: (1) show the seasonal and differential accumulation of ART, DHAA, and AA of elite Brazilian, Chinese, and Swiss cultivars cultivated in a West Virginian field of similar latitude and altitude to Chongqing (China), where approximately 90% of the world's *A. annua* is cultivated for ART extraction; (2) provide evidence that a high-ART genotype can also be high in DHAA, which should be considered as an important sesquiterpene and biochemical marker that can be used in the selection and breeding to produce new high-ART *A. annua* lines.

## Materials and methods

### Plant material and field cultivation

The three main high-ART cultivars used in this study to evaluate seasonal and individual accumulation of sesquiterpenes, were donated by Mediplant (Switzerland, cv. Artemis®), Centro Pluridisciplinar de Pesquisas (CPQBA-Sao Paulo, Brazil, cv. 3M), and Holley Pharma (Chongqing, China, cultivar not identified). Another cultivar (Sandeman Seeds, UK) with high concentration of AA, but low ART and DHAA, was donated by a colleague (Dr. Dae-Kyun Ro). Plants from Brazilian, Chinese, and Sandeman cultivars were started from seeds, while a Swiss selection was cloned in a Quonset greenhouse under long-day photoperiod before transferring to the field. From here on, these genotypes will be mainly referred to as Brazilian, Chinese, Sandeman, and Swiss. For all field experiments reported here, all genotypes were transferred to the field in the first week of June 2006, 2007, or 2009 on an Appalachian soil (Gilpin silt loam—fine-loamy, mixed, mesic Typic Hapludults) at the Richmond School Farm, Beaver, WV (37°45′N 80°50′W, 890 m asl) and provided twice with 45 kg N, 20 kg P, and 37 kg K per hectare during the five-six months of cultivation (June to October/November). Soil pH was 5.8 and plants were irrigated for the first month, until established, with further irrigation only provided by rain. Soil analysis is provided elsewhere (Ferreira, 2007). To determine the seasonal accumulations of ART, DHAA, and AA, Chinese and Brazilian plants were field cultivated in 2006 and 2007, respectively, with three seed-generated plants of each genotype sampled bi-weekly (non-destructively) throughout the season. Seeds of the Swiss genotype (Artemis®), were cultivated in West Virginia and quantified for ART, DHAA, and AA. One selection (named MDP-11) was cloned in 2006 to generate enough plants for the determination of seasonal peak ART. Thus, for the Swiss genotype, three plants of the cloned MDP-11 were harvested at each collection date, the whole plant was oven dried, and a dry sample from the bottom, middle, and top part of each plant was pooled for HPLC-UV analysis. To evaluate the natural segregation of ART, DHAA, and AA in both Swiss and the Brazilian genotypes, approximately 100 plants generated from seeds were transferred to the field, and 55–65 plants were harvested at random on August 24, 2007 (Brazilian) and August 18/19, 2008 (Swiss). Dried leaves were separated from stems, ground to 0.5 mm particle size in a Wiley mill, and saved in a −20°C freezer until extraction for HPLC-UV analysis of underivatized ART and its precursors (Ferreira and Gonzalez, 2009).

### Extraction of ART, its precursors, and HPLC analysis

ART, deoxyartemisinin (co-synthesized with ART), DHAA, and AA were extracted from 500 mg of *A. annua* dry leaf samples, refluxed with 50 mL of petroleum ether (45°C) for 1 h, transferred to beakers and left to dry overnight in a fume hood. Next day, samples were reconstituted in 20 mL of acetonitrile (two washes of 10 mL each), filtered through a 0.45 μm nylon filter attached to a 10-mL luer-lock syringe and transferred to a 20-mL scintillation vial. Samples were transferred to 1.8 mL HPLC vials and 10 μL were injected by an HPLC auto-sampler into the system (Agilent 1100 series). ART, DHAA, and AA were quantified by HPLC-UV (Ferreira and Gonzalez, 2009). Standards of ART were purchased from Sigma/Aldrich (sigmaaldrich.com) and standards of DHAA and AA were donated by Amyris (Amyris.com). To better follow results and discussion, and the roles of ART, DHAA, and AA and their competing pathways, see Figure 1.

### Relationship among sesquiterpenes in *A. annua*

The mathematical model formula used to evaluate ART leaf concentration (ART%) in relation to shoot concentration of AA (AA%) in Figures 4, 5 is shown in Equation 1, and is equivalent to a log-normal distribution fit, where *ln* = Napierian logarithm and *e* is Euler number = 2.1718.

(1) $$\begin{array}{r} ART\left(\%\right)=\frac{0.36\pm 0.06}{AA\left(\%\right)}\times e^{\left(-0.5\times {\left(\frac{\ln\left(\frac{AA\left(\%\right)}{0.34\pm 0.03}\right)}{\ln\left(0.19\pm 0.01\right)}\right)}^{2}\right)} \end{array}$$

## Results

### Differential seasonal sesquiterpene accumulation in *A. annua*

**Figure 2:**
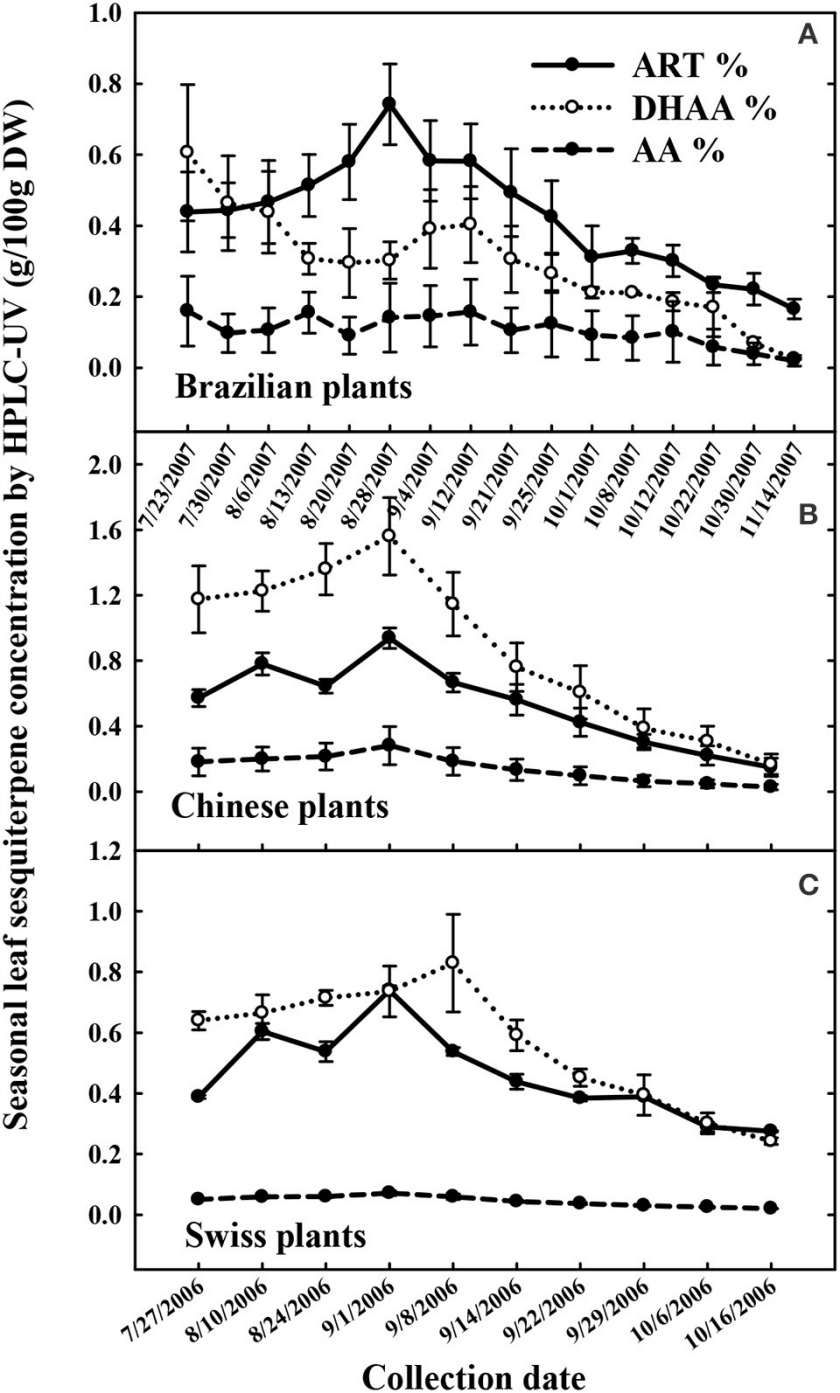
Seasonal accumulation of artemisinin (ART), dihydroartemisinic acid (DHAA), and artemisinic acid (AA) in field cultivated *Artemisia annua* genotypes from Brazil **(A)**, China **(B)**, and Switzerland **(C)** field-cultivated in 2006, and 2007 in West Virginia, USA. All cultivars reached peak ART in late August to early September.

In West Virginia, the seasonal peak of ART concentration for the Brazilian, Chinese, and Swiss cultivars occurred between the end of August and the first week of September, declining steadily thereafter (Figure 2). All Plants of these three cultivars were high in ART and DHAA, but low in AA. The Brazilian cultivar had lower DHAA than ART, the Swiss cultivar had DHAA in similar or slightly higher concentrations than ART, and the Chinese cultivar had higher DHAA than ART. The Chinese cultivar displayed the highest concentrations of DHAA, reaching 1.6% DHAA and 0.95% ART at its seasonal peak (Sept 1). The Swiss plants that were asexually propagated as clones, expectedly showed a small plant-to-plant variation in sesquiterpene content than the Brazilian or Chinese plants generated from seeds (Figure 2). At the peak, the Brazilian and Swiss plants produced an average ART concentration of 0.75%, but a few plants reached 1.5% ART (data not shown). The peak for DHAA concentration coincided with ART in the Chinese and Swiss plants, whereas DHAA was lower than ART during most of the season in Brazilian plants. Plants peaked in their DHAA concentration at 0.6% (July 23, first harvest), 1.58% (Sept 1), and 1.25% (Sept 08) for the Brazilian, Chinese and Swiss plants, respectively. The Brazilian, Chinese, and Swiss genotypes were all low in AA, ranging from 0.1-0.2% and remained fairly constant throughout the whole experiment (Figure 2).

### Sesquiterpene profiling of different *A. annua* chemotypes

**Figure 3:**
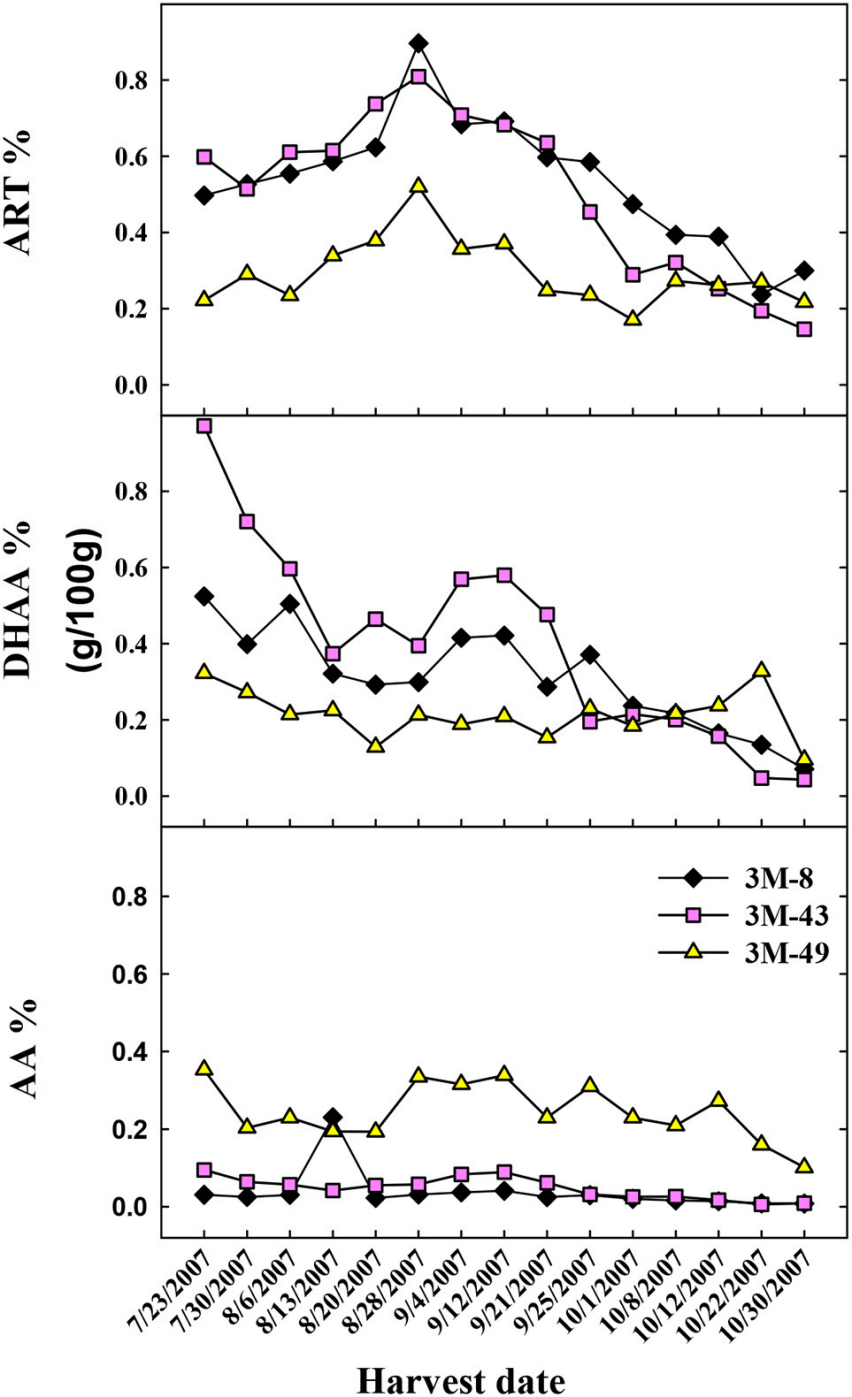
Shoot concentration (g/100 g DW) of the sesquiterpenes, artemisinin (ART%), dihydroartemisinic acid (DHAA%), and artemisinic acid (AA%) in three seed-derived plants from the Brazilian cultivar (3M). Each plant was sampled [**top**, **middle** and **bottom**] weekly and processed for sesquiterpene extraction and HPLC-UV analysis.

Due to the phenotypical segregation observed for DHAA accumulation in 2006, when over 50 seed-generated plants of the Brazilian cultivar were field grown and analyzed (data not shown), three segregating individual plants were cloned and harvested weekly to originate the data in Figure 2. Data for each segregating clone (3M-8, 3M-43, and 3M-49) are shown in Figure 3 regarding the seasonal differences in ART, DHAA, and AA concentrations. Relative concentrations of ART, DHAA, and AA were clearly different among the three genotypes throughout the growing season (Figure 3). ART concentration ranged from 0.2 to 0.9% with maximum and minimum concentrations on August 28th and October 30th, respectively. All three plants peaked in ART concentration on August 28th. Opposite to the Chinese and Swiss genotypes, DHAA content starts high and decreases throughout the growing season in all three Brazilian (3M) clones (Figure 3). AA content was largely unchanged in each of the genotypes throughout the growing season. Genotypes 3M-8 and 3M-43 were most similar in their chemical profiles for all three sesquiterpenes, whereas the 3M-49 displayed lower ART and DHAA content and higher AA content as compared to the other two genotypes for most of the growing season (Figure 3).

In 2008, 50 seed-originated Brazilian and Swiss plants were cultivated in a West Virginia field and in 2009, some seed-originated Sandeman plants were cultivated in the same field. The data for the three elite cultivars and the Sandeman cultivar were grouped for shoot artemisinin concentration (ART %) and artemisinic acid concentrations (AA%) and evaluated through a three-parameter non-linear fit (**Eq. 1**).

**Figure 4:**
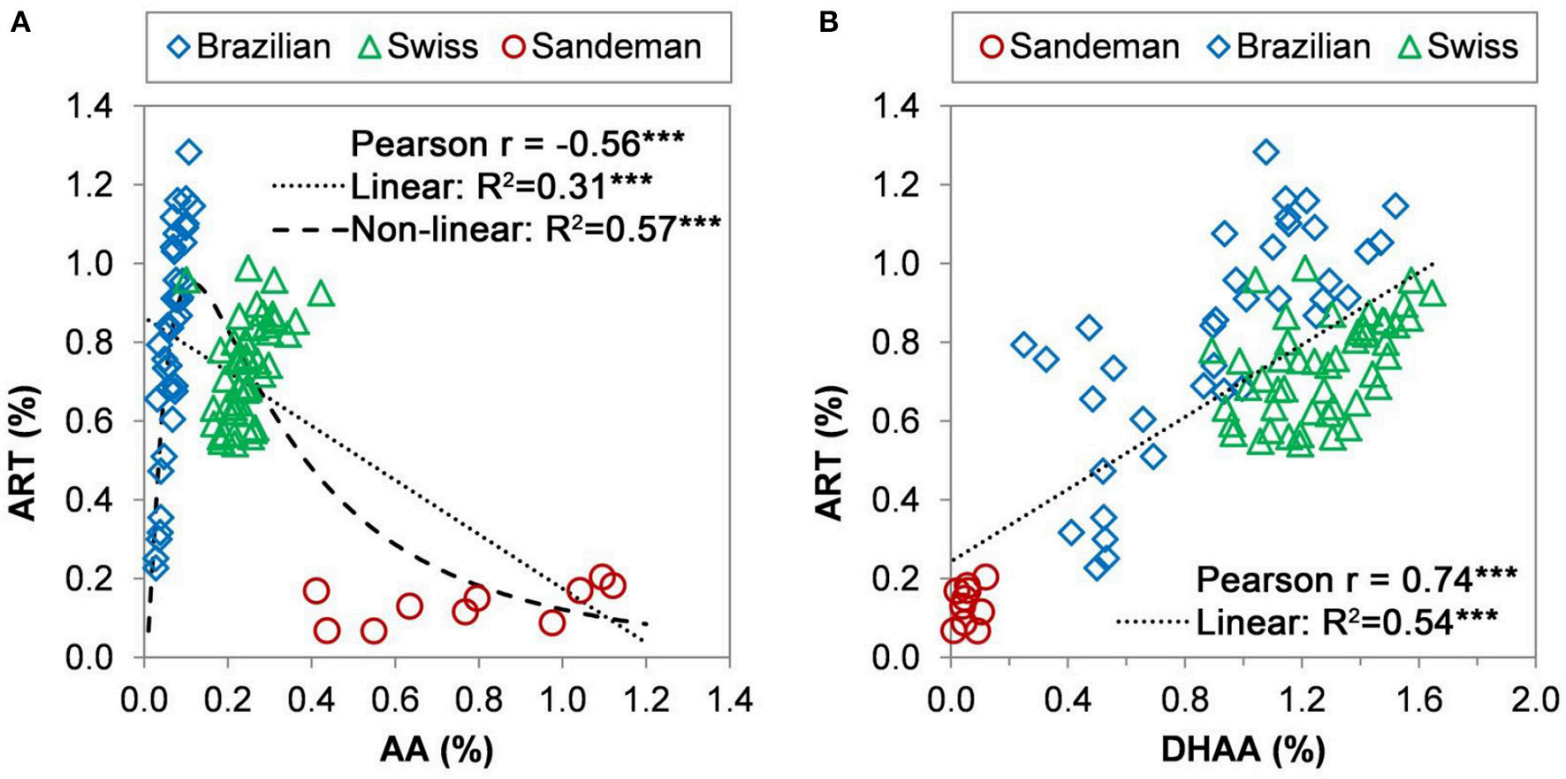
Pearson coefficient correlation between the shoot concentration (g/100 g DW) of dihydroartemisinic acid (DHAA) and artemisinin (ART) (top panels), and of artemisinic acid (AA) and ART (bottom panels) of 38 Brazilian (left) and Swiss (right) seed-derived plants. Plants were grown in the field and collected randomly on August 18–19, 2008, for HPLC-DAD analysis. Correlation between artemisinin and (AA) or (DHAA).

The data were also grouped for leaf ART% and leaf DHAA% and presented as both Pearson (*r, n* = 95) and linear coefficient of determination (*R*^2^, *n* = 95) (Figure 4). When these 95 sexually propagated (37 Brazilian, 48 Swiss, and 10 Sandeman) plants were randomly collected on Aug 18–19, 2008 (Brazilian and Swiss) and 2009 (Sandeman), the Brazilian plants ranged from 0.25 to 1.3% ART, 0.2–1.5% DHAA, and 0.02–0.12 AA, whereas the Swiss plants ranged from 0.58–1% ART, 0.9–1.6% DHAA, and 0.1–0.43 AA, and the Sandeman plants from 0.07–0.2% ART, 0.01–0.12% DHAA, and 0.41–1.18% AA (Figure 4). Data modeling analysis of these 95 plants of four different genotypes (based on leaf sesquiterpene concentrations) resulted in highly significant Pearson (*r* = −0.56^***^, *n* = 95) and a linear coefficient of determination (or fit) of *R*^2^ = 0.31^***^. However, a higher, and highly significant, non-linear fit of *R*^2^ = 0.57^***^ was obtained for the observed *vs*. predicted relationship between ART% vs. AA% (Figure 4A) according to the model in **Eq. 1**. The root mean square error for the ART% predictions, or the diversion of the observed data from the prediction by the model presented in **Eq. 1** was only 0.18%. Interestingly, the range of AA concentration in the sexually propagated progenies was 0.02–0.12% for Brazilian, 0.1–0.43% for Swiss, and 0.41–1.18% for Sandeman plants (Figure 4A).

When correlating ART to DHAA in the three elite cultivars plus the Sandeman plants, both Pearson (*r* = 0.74^***^, *n* = 95) and the linear fit (*R*^2^ = 0.54^***^, *n* = 95) were highly significant (Figure 4B). There was no significant correlation between ART and either AA or DHAA, or between AA and DHAA, for the sexually-propagated Sandeman plants (correlations not shown). The implications of these comparisons across germplasms is explored in the Discussion section.

**Figure 5:**
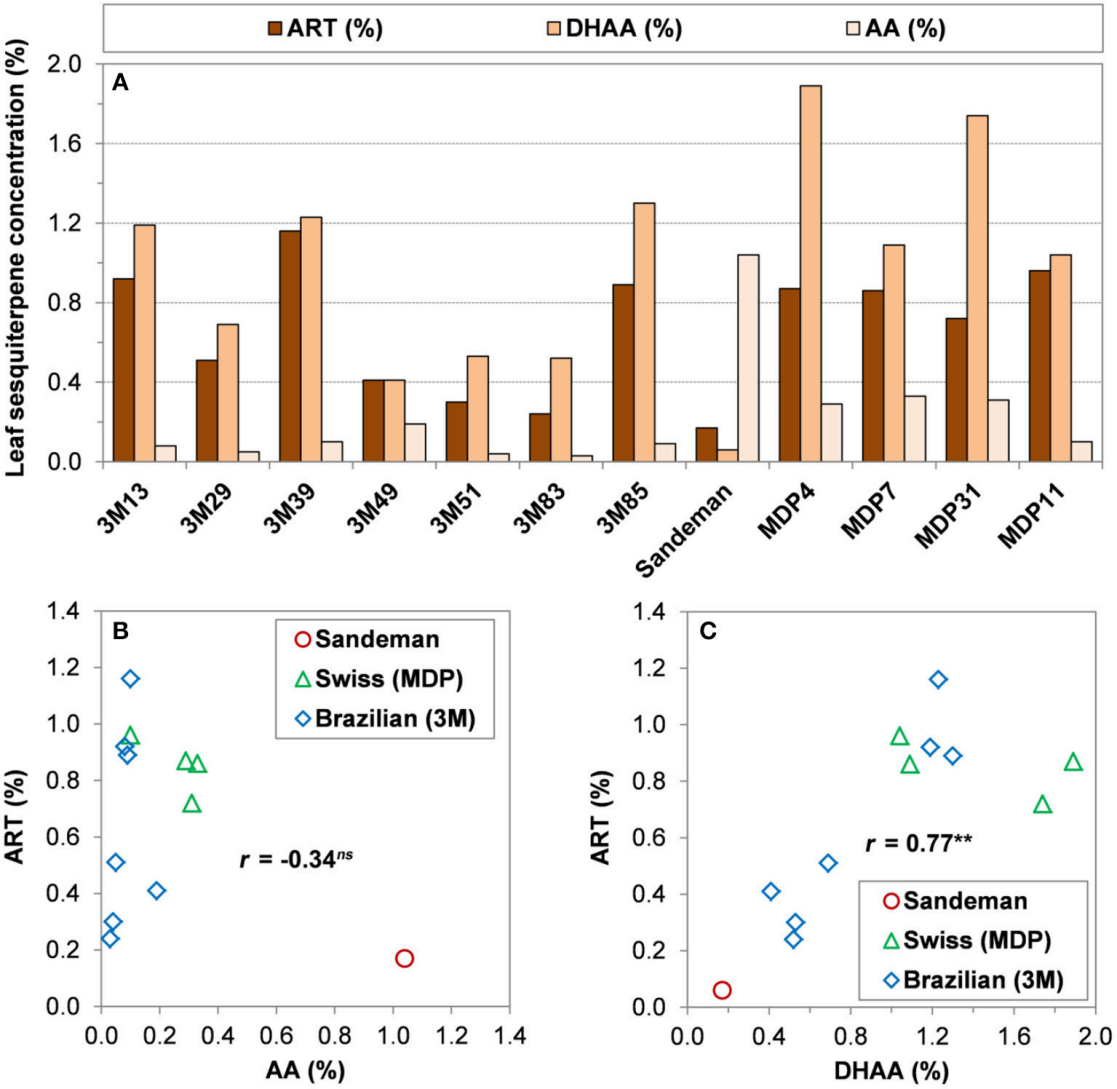
Relationship among three sesquiterpenes present in leaves of different *A. annua* genotypes. **(A)** Leaf concentrations (g/100 g DW) of artemisinin (ART%), dihydroartemisinic acid (DHAA%), and artemisinic acid (AA%) from selected genotypes of Brazilian (3M), Sandeman Seeds UK (Sandeman), and Swiss (Artemis or MDP), maintained at West Virginia University; **(B)** Linear regression between ART% and AA%; **(C)** Linear regression between ART% and DHAA%. Shoot concentrations from greenhouse-grown plants will be slightly higher when grown in the field and collected late August/early September (e.g., 3M-39 = 1.9% ART and MDP 11 = 1.0% ART) in West Virginia.

The several years of HPLC-UV (Ferreira and Gonzalez, 2009) quantification of ART, DHAA, and AA in greenhouse and field-grown plants allowed for a selection of several Brazilian and Swiss clones with contrasting concentrations of all three sesquiterpenes, including a genotype from Sandeman Seeds (UK) that produces over 1% AA and 0.2% or less of either ART or DHAA (Figure 5). The Swiss clones were relatively high in DHAA and ART as compared to the Brazilian and Sandeman plants (Figure 5). For these selected genotypes, DHAA presented significant and high correlation with ART, while AA did not show a significant correlation with ART (Figures 5B,C). Although the Brazilian clones were lower in DHAA than the Swiss clones, three clones that were high in DHAA (3M13, 3M39, and 3M85) were also high in ART (Figure 5A).

## Discussion

Our field data revealed that, regardless of genotype, plants from different origins planted in the same location will reach their ART and DHAA peaks at the same time in the season (Figure 2). The ART peak was not related to flowering and agrees with reports that ART in Swiss plants peaked at the end of August also in Conthey, Switzerland, independently of the physiological state for the hybrid cultivar Artemis® (Delabays et al., 2001). Our results suggest that plants of elite germplasms, regardless of their origins, allocated more resources toward DHAA and ART accumulation, while AA remained below 0.43%, and stable, during the whole season. DHAA biosynthesis was favored in Chinese, similar to ART in Swiss, and lower than ART in Brazilian plants, while AA biosynthesis (opposite to ART or DHAA) was favored in Sandeman plants. Results showed that Brazilian plants produce more ART than DHAA, whereas Chinese plants accumulated more DHAA than ART (Figure 2). Comparing Brazilian to Chinese plants, and accepting the possibility that the conversion of DHAA to ART may involve enzymes (Zhu et al., 2014; Bryant et al., 2015), besides photooxidation, it is possible that the pathway in the Chinese genotype may have enzyme isoforms that are less efficient to convert DHAA into ART, while the Brazilian genotype has isoforms that are very efficient in converting DHAA to ART. A recent report (Czechowski et al., 2018) involving Swiss plants that were either high in ART (Artemis®), or low in ART (NCV) and high in AA, arteannuin B (AB), and artemisitene (AT), confirmed previous (Ferreira, 2007) and our current results of high biosynthesis of ART and DHAA in Swiss plants (Artemis®). Czechowski et al. (2018) remarked that competing pathways had enzymes that produced ART and derivatives, all having a methyl group (CH_3_) on carbon 11, while the other side of the pathway (for AA, AB, and AT) had similar compounds, but with an ethyl group (CH_2_) on carbon 11. These authors hypothesized that the existence of different isoforms of the amorpha-4,11-diene C-12 oxidase (CYP71AV1- steps 2 and 3 of the pathway in Figure 1), may be associated with high- and low-ART plants. They also concluded that the enzyme DBR2 (Step 4, Figure 1) was highly expressed on Artemis® plants that are high in ART and DHAA and that, although no enzyme may be involved, AA somehow converts rather to AB than to AT. Similarly, we have observed during our HPLC-UV analysis that DHAA and DHAAHP, which may (Zhu et al., 2014; Bryant et al., 2015) or may not (Czechowski et al., 2018) be enzyme-substrates, converted preferentially to ART, with very little conversion to dART (Ferreira and Luthria, 2010). Based on this evidence, if we infer that there could be isoforms of aldehyde dehydrogenase (ALDH1) with different efficiencies in converting dihydroartemisinic aldehyde (Step 5, Figure 1) to DHAA, but with low conversion of DHAA to ART, we would have plants such as the Chinese or Swiss (higher DHAA than ART). Alternatively, for high efficiency of conversion of DHAA to ART (higher ART than DHAA), we would have Brazilian plants. Thus, the cross of Brazilian and Chinese would be expected to produce plants with higher ART than either parent.

During the direct quantification of ART and its precursors in the Swiss Artemis® (Ferreira and Gonzalez, 2009), plants produced from 0.65 to 1.9% ART, but no AT or AB. Also, field-grown (Illinois, USA) Brazilian (3M) plants harvested on September 13, 2003 (Peng et al., 2006), produced neither AT or AB, as confirmed by mass spectrometry. These previous results with high-ART Swiss and Brazilian genotypes agree with Czechowski et al. (2018) in that AB and AT may be produced only in low-ART plants, such as their NCV plants (Czechowski et al., 2018).

Considering that plants with high concentrations of DHAA, and low AA, may lead to high ART% in leaves *in planta* or after drying, our data reflects the fact that Chinese, Brazilian, and Swiss plants vary in their accumulation of DHAA and ART, with Brazilian plants having higher ART than DHAA, Swiss plants having similar concentrations of ART and DHAA, and Chinese plants having almost twice as much DHAA as ART (Figure 2). Swiss plants showed a narrower range of both traits compared to the Brazilian clones, and were higher than Brazilian plants in DHAA, while Brazilian plants were higher than the Swiss in ART (Figures 2, 4, and 5). Data from Figure 4 and the sesquiterpene analyses from 63 and 55 seeded plants from Swiss (0.56–1.2% ART) and Brazilian (0.33–1.5% ART) genotypes, respectively, (Supplementary Figures 1, 2) prove that the Brazilian genotype has a broader genetic base compared to the Swiss genotype. This broad base in ART (0.23–0.78% ART) was also previously reported from 16 Brazilian (3M) plants (Peng et al., 2006). This implies that further selection of high-ART Brazilian plants and their crossing with high-DHAA Chinese or Swiss plants may result in progenies with higher ART concentrations than either genotype can currently afford separately. Our data also suggest that these plants should have AA leaf concentrations lower than 0.2% because as AA increases the concentrations of ART and DHAA decrease, as seen for Brazilian plant 3M-49 (Figure 3) and Sandeman plants (Figures 4, 5). Swiss plants had AA over 0.2%, a fair concentration of DHAA and ART, but not as high as Brazilian plants (Figure 4).

Previously, and without knowing their DHAA concentration, Chinese plants have been successfully used as parents to increase ART in Swiss plant developed by Mediplant (Delabays et al., 1993), and that may also explain why these plants have high concentrations of DHAA despite their levels of AA over 0.2% (Figure 5). Later on, the use of Vietnamese plants selected for ART% as high as 1.3%, but with unknown concentrations of DHAA, was also mentioned as a good source of parents to increase ART% in new crosses (Delabays et al., 1995). On the onset of ART research in China, an ART-rich *A. annua*, originally from the Sichuan Province, was used in “Project 523” to afford high-ART extraction (Tu, 2011). It is interesting to note that a recent publication involving a Sichuan Province genotype reported control sesquiterpene concentrations of 0.6% ART, 2% DHAA, and 0.06% AA (Wang et al., 2010). These sesquiterpene ratios are similar to the ones reported in this work for the Chinese genotype (average of 0.9% ART, 1.6% DHAA, 0.02% AA) (Figure 2). We assume that Vietnamese plants, originally imported from China, may also be high in DHAA, and a good parent source to breed high-ART plants. Thus, the differences in ART and DHAA contents found between the Brazilian and both Swiss and Chinese plants (both high in DHAA) can be explored to breed novel genetic recombinants high in ART. The enzymatic ability to convert most of the DHAA into ART can be genetically acquired from the Brazilian plants. The excess DHAA, not converted to ART *in vivo*, could still be converted into ART during drying (Ferreira and Luthria, 2010), suspending the irrigation to stress the plants close to harvest (Marchese et al., 2010), extracted to be converted into ART semi-synthetically (Kopetzki et al., 2013), or extracted from ART commercial waste as raw material for ART semi-synthesis (Liu et al., 2017).

Previous studies proved that the broad-sense heritability was high (*H*^2^ > 0.98) for the ART trait (Ferreira et al., 1995). This was later confirmed by narrow-sense heritability (Delabays et al., 2001) suggesting that additive effects play a major role in the total genetic variance, and that selection for both ART and DHAA traits will be highly and efficiently conserved, leading to further increase in ART leaf concentration of new hybrids. An individual Brazilian clone (3M-49, Figure 3) and 10 seed-generated Sandeman plants (Figure 4) illustrate that a genotype that produces AA as the main sesquiterpene will produce ART and DHAA in low concentrations, and vice-versa, therefore confirming that DHAA/ART and AA are in two competing branches of the biosynthetic pathway and should be considered for their additive (ART and DHAA) or negative (AA) effects when breeding new high-ART genotypes. Data generated from the 10 Sandeman-UK seeded plants clearly shows that plants with high leaf concentrations of AA should not be used to generate new genotypes high in ART and DHAA. Confirming this line of thought, a previous attempt to select high-ART clones using tissue culture-, greenhouse-, and field-propagated plants of unknown origin produced plants with 0.4 to 1.0% AA, but with only 0.03–0.06% ART (Elhag et al., 1992). Their *A. annua* plants, field-cultivated in Saudi Arabia, produced a very similar picture to that reported in our work for Sandeman plants cultivated in West Virginia in 2009 (0.41–1.18% AA, 0.01–0.12% DHAA, and 0.07–0.2% ART, Figure 4). Although reported from different sites and in different years, these similar results attest to the fact that these populations, possibly unrelated, maintain their characteristic pathways leading to plants that are mostly high in AA and very low in ART and DHAA.

Our yearly records for the Brazilian and Swiss clones strongly support that plants high in ART and DHAA are lower in AA and vice-versa and individual plant sesquiterpene profiles and Pearson coefficients in Figure 5 (*r* = −0.34^ns^ for AA vs. ART and *r* = 0.77^**^ for DHAA vs. ART, *n* = 12) mirror the sesquiterpene profile and coefficients of their seed-generated 95 plants from the same germplasm (*r* = −0.56^***^ for AA vs. ART and *r* = 0.74^***^ for DHAA vs. ART, *n* = 95) in Figures 4A,B. The highly significant non-linear fit (*R*^2^ = 0.57^***^) obtained between AA% and ART% reflects the fact that Brazilian plants were higher in ART but had a very low leaf concentration of AA. As the AA% rises to 0.2% or higher (Swiss plants), ART started to decrease and when AA% was from 0.4 to 1.18% (Sandeman plants), leaf ART% was never higher than 0.2% (Figure 4). Again, these Pearson coefficients suggest a negative correlation between AA and ART (*r* = −0.56^***^ and −0.34^ns^, Figures 4, 5, respectively) as they are biosynthesized in competing sides of the same pathway.

An *A. annua* germplasm collection encompassing twelve genetically diverse clones (Figure 5) was established at the USDA-ARS Appalachian Farming Systems Research Center, Beaver, WV was clonally propagated and maintained for approximately five years at the USDA-ARS in Beaver, and is currently maintained at the Evansdale Greenhouse (West Virginia University, Morgantown, WV). This collection holds clones with distinct chemotypes from Brazil and Switzerland, plus a clone originated from a commercial nursery (Sandeman Seeds, UK) that is high in AA, but low in ART and DHAA (Figure 5). These plants have maintained their concentrations of the sesquiterpenes they were selected for.

For the ten plants generated from Sandeman seeds, the lack of significant correlation between any of the sesquiterpenes reflects the fact that AA is not a precursor of ART, but may be a precursor of AB and AT in its side of the pathway (Figure 1). Plants high in DHAA may produce even higher ART yields if stressed by abiotic factors such as drought, potassium deficiency, and salinity, or during commercial drying, as previously reported (Brown and Sy, 2004; Ferreira, 2007; Ferreira and Luthria, 2010; Marchese et al., 2010; Yadav et al., 2017). However, more research is needed to determine the extent, and which stress may increase ART without compromising biomass yield. In the meanwhile, new different systems for the *in vitro* conversion of DHAA into ART or its antimalarial derivatives (e.g., artemether and artemotil) have been recently reviewed and reported encouraging yields of up to 65% (Lévesque and Seeberger, 2012; Howard et al., 2017).

Remarkably, the step involved in converting DHAA into ART is thought to be mediated by photooxidation instead of through an enzymatic catalysis (Brown, 2010; Czechowski et al., 2018). This suggests that the photooxidation step may be differentially regulated by an unknown mechanism between the Chinese and Brazilian plants. Alternatively, as the conditions for chemical reaction used for semi-synthetic synthesis of ART may not exist in plants, there is the possibility that an unknown biochemical process is involved in the DHAA conversion to ART (Xie et al., 2016) and, surprisingly, it could even involve an enzyme *in vivo* (Zhu et al., 2014; Bryant et al., 2015). However, one should keep in mind that the reported non-enzymatic conversion of DHAA to ART had only a 23% overall yield (Acton and Roth, 1992) and that even with optimized conditions and the addition of 0.24 nmol of chlorophyll *a*, it took 129 h to convert 26.8% of the DHAA into ART (Wallaart et al., 1999b). However, drying plant material for 12–16 h in a forced-air oven (in the dark) at 40°C allowed an estimated 40% conversion of DHAA to ART, while sun drying from 1 to 3 weeks resulted in a 94% conversion (Ferreira and Luthria, 2010). Also, suspending irrigation in field plants 38 h before harvest resulted in an ART increase of 29% compared to the irrigated control (Marchese et al., 2010). The higher conversions achieved by oven, drought and sun drying, compared to conversions in organic solvents reported by Acton and Wallaart's research teams, indicate that either ROS produced by heat stress are rapidly being used to convert DHAA into ART or that the conversion could, at least partially, involve enzymes. Freeze dried plant samples had significantly higher concentrations of DHAA and lower concentrations of ART (Ferreira and Luthria, 2010) as if the conversion had been blocked by either the freezing of enzymes or by the absence of oxygen during the freeze drying process, or both. A similar low concentration of ART was reported for both freeze drying and microwave drying compared to open-air drying (Ferreira et al., 1992). In addition, oven drying leaves at 20–40°C, for at most 25 h of drying, did not alter artemisinin concentration in dried leaves, while drying at 90°C for 5 hours significantly decreased ART% in leaves (Xavier Simonnet, personal communication). Thus, it seems that although some conversion of DHAA to ART can happen without enzymes, it does not rule out the participation of enzymes that could speed up the conversion of DHAA into ART as the plant dies, which could work simultaneously with photooxidation to assure the highest amount of ART in glandular trichomes during plant death.

## Conclusions

Our field data shows for the first time that, regardless of the genotype, plants consistently reach their ART peaks during the same approximate time of the year, toward the end of their vegetative stage, suggesting that the ART is related to photoperiod, not flowering, and confirming reports for peak ART in vegetative Swiss plants (Artemis®) cultivated in Conthey, Switzerland. Over the years, this seasonal peak remained true regarding the sesquiterpene profile of cloned plants maintained in a greenhouse under long days during the winter. This propagation strategy and reliable ART concentration were also used by an *A. annua* breeding program at Rutgers University that used one of our Brazilian (3M) selections from Illinois (USA), which was reported to produce 1.5% ART (Wang et al., 2005). Plants left in the field flowered and died as days got shorter in the winter. Our results reiterate previous reports that ART and DHAA compete for substrate with the AA side of the pathway, and that DHAA is the main precursor of ART in high-artemisinin chemotypes. Although concentrations of AA may correlate to ART concentration in the same cultivar, it remained low and unchanged throughout the season in all cultivars studied for seasonal ART peak. Thus, for breeding purposes, using a genotype high in AA will not lead to plants high in ART or DHAA as previously published (Elhag et al., 1992), and confirming our results with the Sandeman cultivar. Field studies with several seed-originated plants of selected Brazilian genotype (3M, CPQBA) and Swiss genotype (Artemis®, Mediplant) (Figure 4) indicated that the Swiss hybrids have parents with an allelic composition less diverse related to the ART trait than Brazilian plants. Both, Brazilian and Swiss plants varied in their ART and DHAA contents, but homogeneously produced plants with as much as 1.8% DHAA (Swiss, Supplementary Figure 1) and 1.5% ART (Brazilian, Supplementary Figure 2) and that were either higher in ART than DHAA (Brazilian) or vice-versa (Chinese and Swiss). Chinese plants had the highest levels of DHAA and these plants could provide suitable alleles for breeding, as they also contributed to generate the Swiss cultivar Artemis® in 1999. Our results confirmed the reports by Wallaart et al. (2000) that cultivars that have higher concentrations of DHAA than ART are low in AA, and vice-versa, recently also confirmed by Czechowski et al. (2018). Plants that have higher DHAA than ART (Chinese) may be crossed with plants that have higher ART than DHAA (Brazilian) to generate plants with even higher ART than either parent, as these genotypes may have an ideal combination of enzymatic systems needed to transform DHAA into ART. Although the cross could not be genetically confirmed, but based on their low selfing rate, crosses between Brazilian and Chinese plants, done by pairing plants synchronized to flower in Indiana (Purdue), and selected in Georgia were recently reported to produce 2.16% ART (Wetzstein et al., 2018). Alternatively, genotypes with high DHAA may be genetically engineered to overexpress enzymes needed to convert DHAA into ART, if eventually demonstrated that indeed enzymes are involved in the end of the ART pathway *in vivo* (Zhu et al., 2014).

Based on the evidence provided by several years of metabolomics study of three elite germplasms of *A. annua* and by cross referencing of previous published literature, this work presents strong evidence and a novel approach that suggests that future *A. annua* breeding programs should consider plants of different chemotypes that are high in both, ART and DHAA and low in AA. If the *in planta* conversion of DHAA into ART is indeed non-enzymatic and only requires photooxidation, the role of ROS in this conversion and the use of both light and oxygen during either pre-harvest water stress or post-harvest drying may prove to be useful to increase leaf concentration of ART. Finally, plants that are high in DHAA can also provide this compound as a precursor for the semi-synthesis of ART in continuous flow systems that have so far proved efficient for the *in vitro* conversion of DHAA into ART.

## Author contributions

JF conceived the experiments involving seasonal sesquiterpene accumulation and relationship, based on HPLC analysis of sesquiterpenes and participated actively in all drafts of the manuscript. JF, VB, and DS collaborated in the discussion of results and potential use of elite germplasms high in ART and DHAA to increase artemisinin. VB has propagated and kept the germplasm selections presented in Figure 5 for the past 6 years. JM contributed with critical comments in most drafts of the manuscript. SL read and commented on the initial drafts.

### Conflict of interest statement

The authors declare that the research was conducted in the absence of any commercial or financial relationships that could be construed as a potential conflict of interest.

The reviewer TL and handling editor declared their shared affiliation.

## Supplementary material

Supplementary Figure 1 Sesquiterpene profile of Swiss cultivar (“Artemis,” Mediplant) in 63 seed-generated plants, field-cultivated in West Virginia, and harvested on 08/18/2008. All plants contained higher dihydroartemisinic acid (DHAA) in shoots than artemisinin (ART). Subplots show correlation between ART and DHAA concentrations in g/100g DW (%).

Supplementary Figure 2 Sesquiterpene profile of Brazilian cultivar (3M, CPQBA) in 55 seed-generated plants cultivated in field. The majority of the plants contained higher artemisinin (ART) in shoots than dihydroartemisinic acid (DHAA). Subplots show correlation between ART and DHAA concentrations in g/100g DW (%). Concentration of AA for plants 3M-49 (0.28%) and 3M-49 (0.4%), harvested on August 20 and August 28, respectively, were removed not to throw off correlation as they were higher in AA than all the other plants.
