# Supplementary figures and images for: Seasonal and Differential Sesquiterpene Accumulation in Artemisia annua Suggest Selection Based on Both Artemisinin and Dihydroartemisinic Acid may Increase Artemisinin in planta
Source: Front Plant Sci. 2018 Aug 13;9:1096. doi: 10.3389/fpls.2018.01096 (PMC6102481; doi:10.3389/fpls.2018.01096)

**Supplemental data**


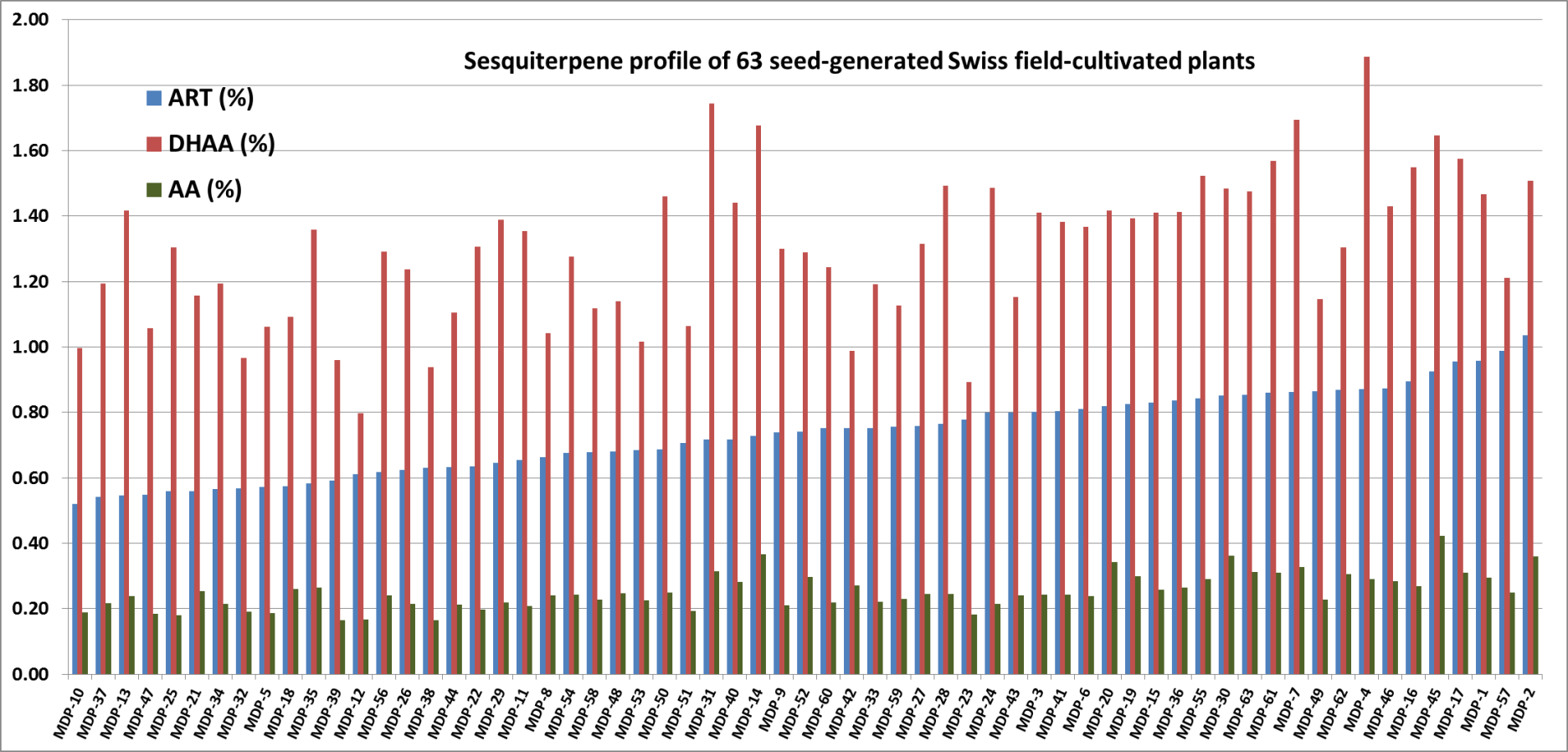


**
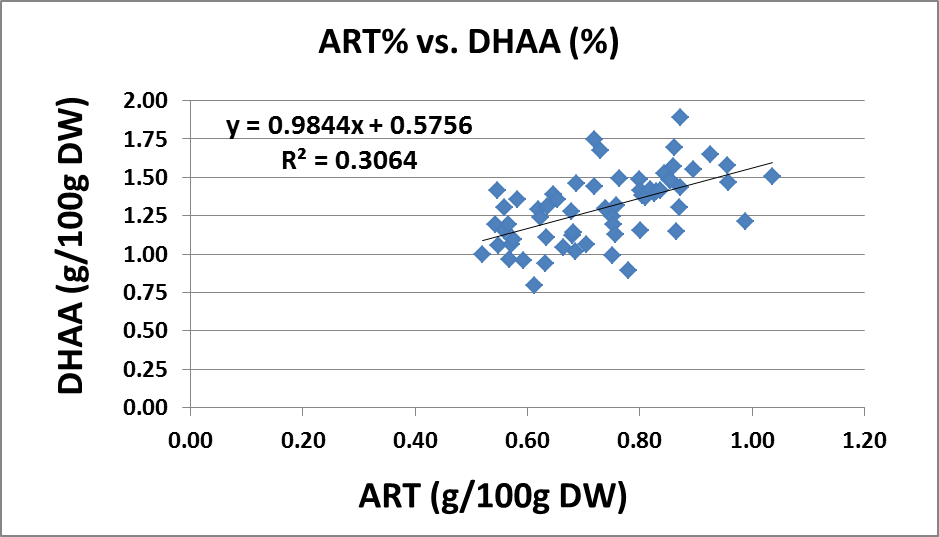

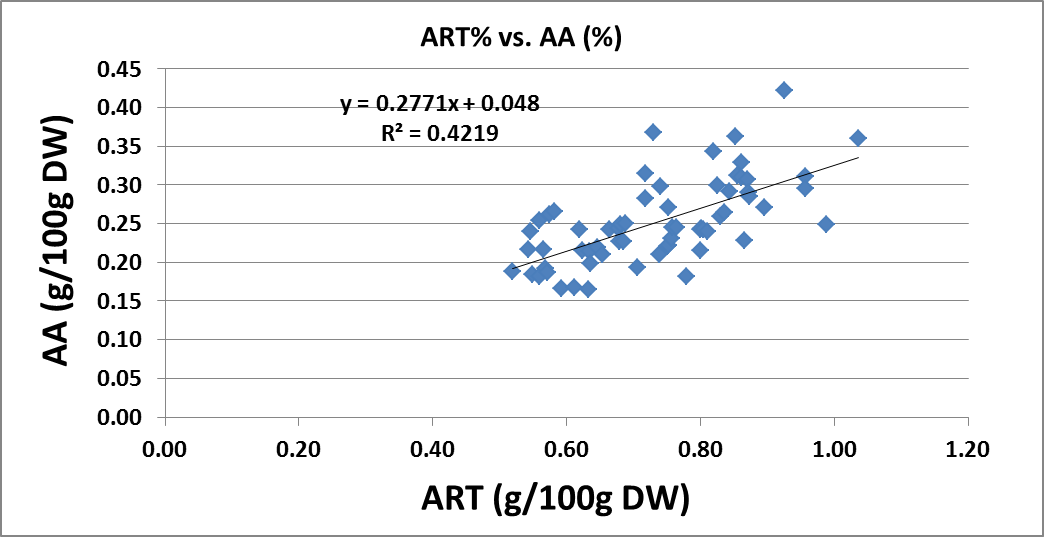
**

**Supplemental Figure 1**

**
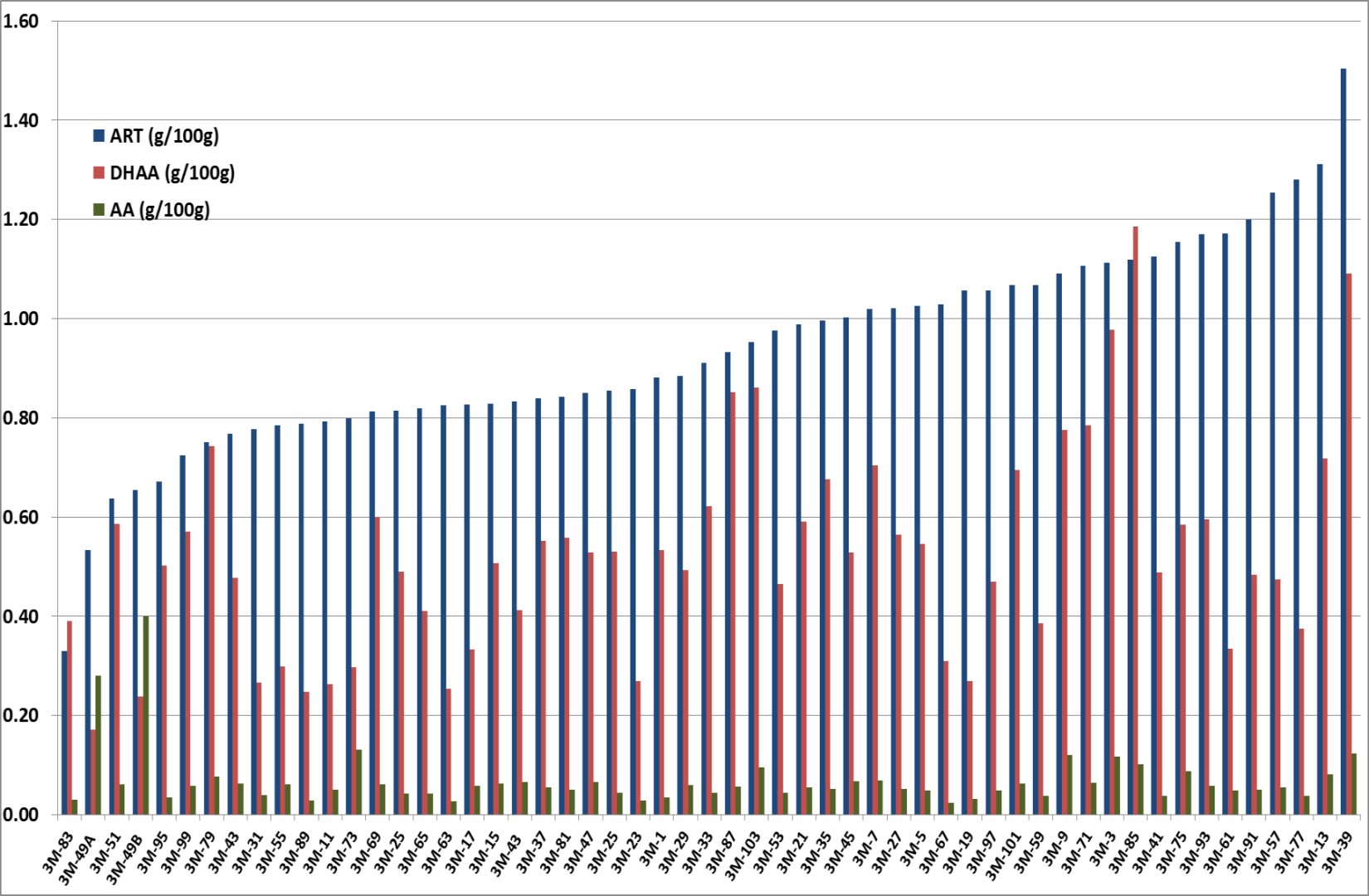
**

**
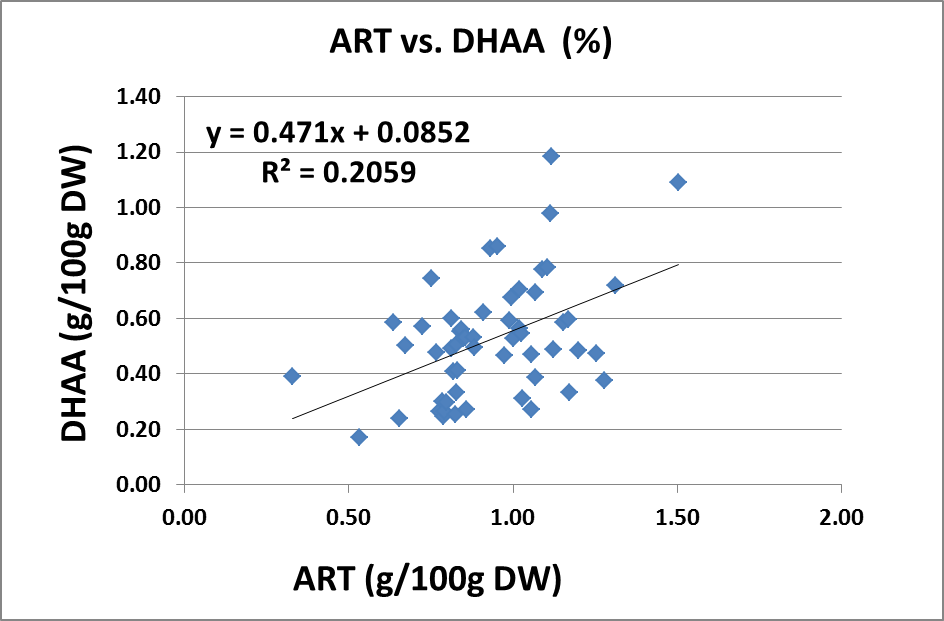

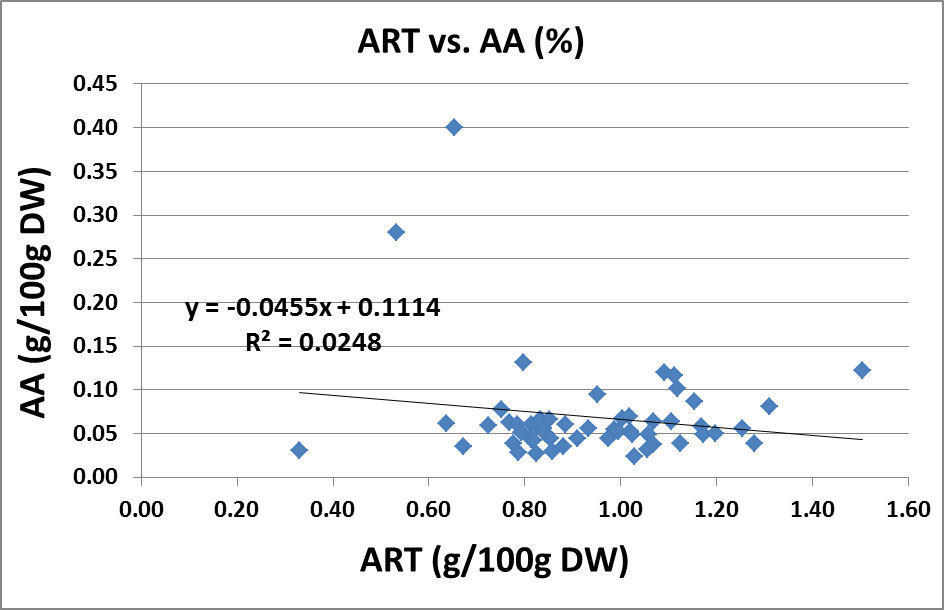
**

**Supplemental Figure 2**

Supplement: Supplementary Figure 1 — Sesquiterpene profile of Swiss cultivar (“Artemis,” Mediplant) in 63 seed-generated plants, field-cultivated in West Virginia, and harvested on 08/18/2008. All plants contained higher dihydroartemisinic acid (DHAA) in shoots than artemisinin (ART). Subplots show correlation between ART and DHAA concentrations in g/100g DW (%). [file Data_Sheet_1.docx]
